# Long-Term Immune Response Profiles to SARS-CoV-2 Vaccination and Infection in People with Multiple Sclerosis on Anti-CD20 Therapy

**DOI:** 10.3390/vaccines11091464

**Published:** 2023-09-07

**Authors:** Christina Woopen, Marie Dunsche, Georges Katoul Al Rahbani, Anja Dillenseger, Yassin Atta, Rocco Haase, Catarina Raposo, Rosetta Pedotti, Tjalf Ziemssen, Katja Akgün

**Affiliations:** 1 Center of Clinical Neuroscience, University Hospital Carl Gustav Carus, Dresden University of Technology, 01307 Dresden, Germany; christina.woopen@uniklinikum-dresden.de (C.W.); tjalf.ziemssen@uniklinikum-dresden.de (T.Z.); 2 F. Hoffmann-La Roche Ltd., 4070 Basel, Switzerland

**Keywords:** SARS-CoV-2, vaccination, anti-CD20 therapy, multiple sclerosis, ocrelizumab, interferon-gamma release assay

## Abstract

Our objective was to analyze longitudinal cellular and humoral immune responses to severe acute respiratory syndrome coronavirus 2 (SARS-CoV-2) vaccination in people with multiple sclerosis (pwMS) on B-cell depleting treatment (BCDT) compared to pwMS without immunotherapy. We further evaluated the impact of COVID-19 infection and vaccination timing. PwMS (*n* = 439) on BCDT (ocrelizumab, rituximab, ofatumumab) or without immunotherapy were recruited for this prospective cohort study between June 2021 and June 2022. SARS-CoV-2 spike-specific antibodies and interferon-γ release of CD4 and CD8 T-cells upon stimulation with spike protein peptide pools were analyzed at different timepoints (after primary vaccination, 3 and 6 months after primary vaccination, after booster vaccination, 3 months after booster). Humoral response to SARS-CoV-2 was consistently lower whereas T-cell response was higher in patients with BCDT compared to controls. Cellular and humoral responses decreased over time after primary vaccination and increased again upon booster vaccination, with significantly higher antibody titers after booster than after primary vaccination in both untreated and B-cell-depleted pwMS. COVID-19 infection further led to a significant increase in SARS-CoV-2-specific responses. Despite attenuated B-cell responses, a third vaccination for patients with BCDT seems recommendable, since at least partial protection can be expected from the strong T-cell response. Moreover, our data show that an assessment of T-cell responses may be helpful in B-cell-depleted patients to evaluate the efficacy of SARS-CoV-2 vaccination.

## 1. Introduction

Multiple sclerosis (MS) is the most common chronic inflammatory disease of the central nervous system; it affects 2.8 million people worldwide and it is the most frequent neurological cause of disability in young adults [1]. 

There are some effective disease-modifying therapies (DMTs) that enable a reduction in disease activity. However, certain DMTs, like anti-CD20 monoclonal antibodies such as ocrelizumab, rituximab, and ofatumumab, may cause an increased risk of severe courses of infection, including that of COVID-19. Several real-world studies conducted during the pre-vaccination era of the pandemic suggest that pwMS treated with anti-CD20 agents have an increased risk of COVID-19-related hospitalization. Vaccination and the generation of protective immune responses against SARS-CoV-2 are of paramount importance for this vulnerable cohort. Many studies have shown decreased humoral responses to SARS-CoV-2 primary vaccination (two doses of vaccines) in anti-CD20-treated patients, whereas T-cell responses were similar or even enhanced in comparison to healthy controls or untreated patients in most studies [2,3,4,5,6,7,8,9,10,11,12,13,14,15,16,17]. While immune responses to primary vaccination are relatively well studied, less is known about the durability of these vaccination responses. Similar to the kinetics of antibodies in the general population, several studies showed a decrease in SARS-CoV-2-specific humoral responses during the six months after initial vaccination in anti-CD20-treated people with MS (pwMS) [17,18,19,20,21,22], whereas other studies reported no significant reduction in antibody titers over time, possibly attributable to initially low titers [3,23,24,25,26]. As for virus-specific T-cell responses, two studies reported a decline thereof in the months following primary vaccination [16,18]. A different study reported higher T-cell responses in ocrelizumab-treated patients 36 weeks after SARS-CoV-2 vaccination compared to other DMTs [17].

Data on the effect of booster vaccination (third dose) in anti-CD20-treated pwMS are also inconclusive. As for humoral responses, multiple studies demonstrated a significant increase in SARS-CoV-2-specific antibodies after booster vaccination in anti-CD20-treated pwMS, albeit with a less pronounced increment compared to healthy controls or pwMS receiving other DMTs [19,21,22,25,27,28]. Contrarily, two studies did not show a significant increase in antibody titers after booster vaccination in ocrelizumab-treated pwMS [3,29]. Concerning T-cell responses to SARS-CoV-2 vaccination, several studies demonstrated an increase of virus-specific T-cells in ocrelizumab-treated pwMS after the booster vaccination compared to the last measurement before the booster [16,18,28]. In contrast, another study reported similar levels of SARS-CoV-2-specific T-cell responses before and after the booster vaccine shot [30].

In order to contribute to a clearer understanding of vaccine response durability and booster effects under anti-CD20 treatment, we assessed the humoral and T-cellular immune response to SARS-CoV-2 vaccination and COVID-19 infection, its durability, and development after booster vaccination in pwMS under anti-CD20 treatment compared to pwMS without DMTs. Our study comprises data from a relatively large number of patients in a real-world cohort of pwMS with a longitudinal follow-up of both humoral and cellular immune responses after primary and booster vaccinations and COVID-19 infection, and therewith offers additional evidence as a basis for clinicians’ vaccination recommendations.

## 2. Materials and Methods

### 2.1. Study Design and Participants

For this prospective cohort study, we recruited patients with a diagnosis of chronic demyelinating disease of the central nervous system during routine clinical visits at the MS Center Dresden, Germany, who had undergone SARS-CoV-2 vaccination. Recruitment took place between 14 June 2021 and 8 June 2022. Patients were eligible for recruitment if they had a diagnosis of MS and were older than 18 years. In this interim analysis, we included only patients who received a B-cell-depleting therapy (BCDT; ocrelizumab, rituximab, or ofatumumab) or had no immunomodulatory treatment. The usual treatment regimen for ocrelizumab was intravenous infusions every six months at a dose of 600 mg, for rituximab intravenous infusions every six months at a dose of 500 mg or 1000 mg, and for ofatumumab subcutaneous injections at a dose of 20 mg every four weeks. Patients who discontinued anti-CD20 therapy remained in the same subgroup in our analysis. Patients without immunomodulation were either treatment-naïve or had their previous DMT dose at least 28 days before vaccination. Patients with a positive anti-SARS-CoV-2 T-cellular or humoral response in the first analysis were scheduled for a retesting of both T-cell and humoral response to SARS-CoV-2 during their next routine clinical visit three or six months after the first analysis, respectively.

### 2.2. Assessment of T-Cellular Responses to SARS-CoV-2

For the analysis of T-cell responses, lithium heparin blood was collected. As described before, interferon-γ secretion of CD4 and CD8 T-cells was assessed with the SARS-CoV-2 QuantiFERON test (Qiagen, Hilden, Germany) after incubation of blood samples with two SARS-CoV-2 spike protein antigen pools for 16 to 24 h [11]. We measured two technical replicates each for responses to antigen pools 1 and 2, negative, and mitogen controls. Interferon-γ release to antigen pool 1 represents the cytokine secretion of CD4 T-cells, and interferon-γ secretion to antigen pool 2 demonstrates CD4 and CD8 T-cell response. For statistical analysis, the mean of the replicates of the negative controls was subtracted from the mean of the replicates of antigen 1 and 2 responses, respectively. Values above 0.15 IU/mL were defined as positive according to the manufacturer’s instructions. The upper detection limit was 8.4 IU/mL. Values > 8.4 IU/mL were set to 8.5 IU/mL.

### 2.3. Assessment of Humoral Responses to SARS-CoV-2

For the analysis of humoral responses, serum was collected from all participants. IgG antibodies specific for the receptor binding domain (RBD) of the SARS-CoV-2 spike protein were measured by means of electrochemiluminescence immunoassay on a COBAS e801 module (Roche, Basel, Switzerland). Values above 0.8 U/mL were considered positive as suggested in the manufacturer’s instructions. Values below the lower detection limit of 0.4 U/mL were set to 0.2 U/mL, values above the upper detection limit of 25,000 U/mL were set to 25,001 U/mL. The unit U/mL is equivalent to the WHO international standard BAU/mL and does not require conversion [31].

### 2.4. Statistical Analysis

Normal distribution of data was visually assessed using quantile-quantile plots. Results were presented as mean, standard deviation (SD), or 95% confidence intervals (CI). Data were analyzed applying generalized linear mixed models (GLMM) with a linear link function for normally distributed data and gamma distribution and a log link function for right-skewed data. Sex, age, Expanded Disability Status Scale (EDSS) as measure of disability, time between vaccination and blood sampling, timepoint, type of vaccination, confirmed previous COVID-19 infection, DMT group, and the interaction of DMT group and timepoint served as fixed factors. *p*-values < 0.05 were considered statistically significant. For pairwise comparisons, contrast tests with Sidak correction were applied. For additional group comparisons, Chi-Squared tests and Mann–Whitney-U tests were used. Spearman’s correlation was used for the assessment of potential correlations. Statistical analyses were performed using the IBM SPSS software (version 28.0, IBM Corporation, Armonk, NY, USA) and data were visualized using GraphPad Prism (version 8; GraphPad Software, La Jolla, CA, USA).

## 3. Results

### 3.1. Patient Characteristics after Primary Vaccination

A total of 368 patients were included in our analyses after primary vaccination (Table 1). Mean age was 46.4 years; 257 patients (69.8%) were female. Most of the patients presented a relapsing-remitting (77.7%) or progressive MS disease course (12.8%). Primary vaccination consisted of two SARS-CoV-2 vaccine doses in 354 (96.2%) patients. Fourteen patients (3.8%) met the criteria of primary vaccination by the combination of one vaccination and one active SARS-CoV-2 infection. Two-hundred-and -eighty-five patients (77.4%) were treated with ocrelizumab, two (0.5%) with rituximab, and two (0.5%) with ofatumumab. Seventy-nine (21.5%) patients received no immunomodulatory treatment. The mean time period between primary vaccination and blood testing was 127.34 (64.09) days (mean (SD)). At primary vaccination, patients on BCDT had an average time between vaccination and last infusion of 107.5 days, as well as a shorter time period between blood sampling and vaccination (121.2 vs. 150.5 days, *p* = 0.002) and a higher proportion of male patients (33.3% vs. 18.2%, *p* = 0.12) compared to the untreated patient group.

### 3.2. SARS-CoV-2-Specific T- and B-Cellular Immune Response after Primary Vaccination

**Figure 1:**
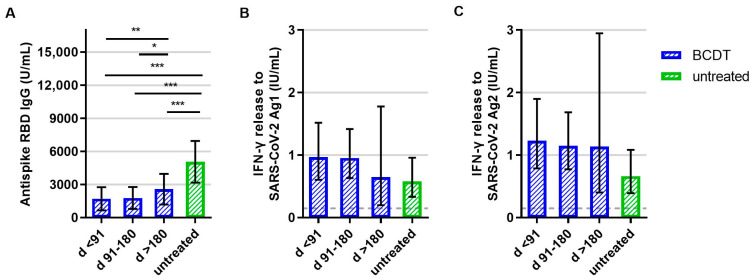
B- and T-cellular responses to primary vaccination in anti-CD20-depleted versus untreated patients (*n* = 348). B- and T-cell responses in patients with B-cell-depleting therapy (BCDT) (blue, *n* = 271) and untreated (green, *n* = 77) patients to the primary vaccination are presented. BCDT is further differentiated regarding timepoint of last BCDT treatment cycle and vaccination: <91 days (*n* = 95), between 91 and 180 days (*n* = 165) and >180 days (*n* = 11) before vaccination. (**A**) presents the B-cell response, (**B**) the T-cell response to Ag1, and (**C**) the T-cell response to Ag2. Means with 95%CI are presented. The dashed line shows the cut-off for a positive T-cell response, defined at 0.015 IU/mL. For the B-cell response, the cut-off level is defined at 0.08 U/mL. Data were analyzed with generalized linear models with gamma distribution and log link function. Sex, age, Expanded Disability Status Scale, time between vaccination and blood sampling, confirmed previous COVID-19 infection, and treatment group served as fixed factors. For pairwise comparisons, contrast tests with Sidak correction were applied. Asterisks indicate level of statistical significance: * *p* < 0.05, ** *p* < 0.01, *** *p* < 0.001. (**A**): *p* < 0.001 for untreated compared to d > 180, *p* < 0.001 for untreated compared to d 91–180, *p* < 0.001 for untreated compared to d < 91, *p* = 0.008 for d > 180 compared to d < 91, *p* = 0.013 d > 180 compared to d 91–180.

Analyses of immune responses after primary vaccination showed that patients with an anti-CD20 therapy presented lower anti-RBD antibody titers compared to patients without treatment (Figure 1A). Moreover, patients with a longer time period (91 to 180 days or more than 180 days) between the last treatment cycle and SARS-CoV-2 vaccination had higher antibody titers than the ones who had received the first vaccination less than 90 days after their last treatment (Figure 1A). Concerning the T-cell response, there was a trend for an increased IFN-γ release in anti-CD20-treated patients compared to patients without treatment. However, differences between the groups were not significant (*p* = 0.139 for Ag1, *p* = 0.061 for Ag2; Figure 1B,C).

**Figure 2:**
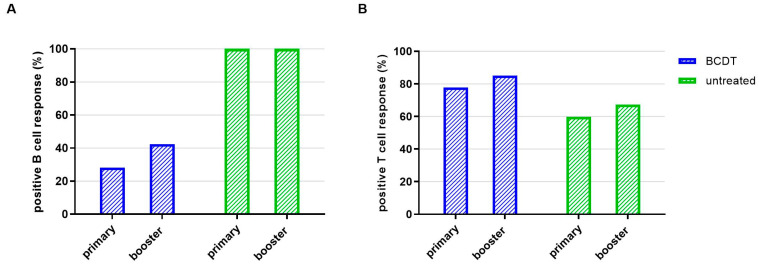
Seroconversion rate of B- and T-cellular responses in anti-CD20 depleted versus untreated patients (*n* = 367). Seroconversion rate regarding B- (**A**) and T- (**B**) cell responses is presented for patients with B-cell-depleting therapy (blue, *n* = 270) and untreated patients (green, *n* = 77).

Evaluating the percentage of patients that presented B- and T-cell response levels above the predefined cut-off, 28.2% of the patients with anti-CD20 therapy had a detectable SARS-CoV-2-antibody response after primary vaccination and 77.7% had a positive T-cell response. In contrast, 100% of patients without treatment had a positive B-cell response and 59.7% positive T-cell response after primary vaccination (Figure 2, for all group comparisons: *p* < 0.05).

**Figure 3:**
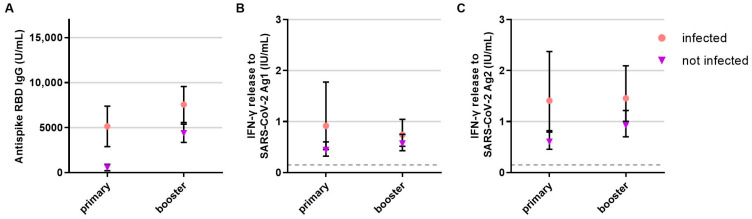
Influence of COVID-19 infection on the B- and T-cellular responses to primary and booster vaccination in anti-CD20 depleted versus untreated patients (*n* = 341). B- and T-cell responses to primary and booster vaccination with additional COVID-19 infection (pink, circle, respective *n* for each time point: 244, 248) or without COVID-19 infection (purple, triangle, respective *n* for each time point: 32, 51) are presented. (**A**) B-cell response, (**B**) T-cell response to Ag1, (**C**) T-cell response to Ag2. Means with 95%CI are depicted. The dashed line shows the cut-off for a positive T-cell response, defined at 0.015 IU/mL. For the B-cell response, the cut-off level is defined at 0.8 U/mL. Data were analyzed with generalized linear mixed models (GLMM) with gamma distribution and log link function. Sex, age, Expanded Disability Status Scale as measure of disability, time between vaccination and blood sampling, timepoint, type of vaccination, confirmed previous COVID-19 infection, treatment group, and the interaction of treatment group and timepoint served as fixed factors. For pairwise comparisons, contrast tests with Sidak correction were applied.

The number of SARS-CoV-2 infections (pre-vaccination or breakthrough infection) was similar in patients on B-cell-depleting therapy and without DMT (Table 2). Infections had a significant effect on RBD antibody titers (*p* < 0.001) and T-cell responses after primary vaccination (*p* = 0.001 for Ag1 and *p* < 0.001 for Ag2, Figure 3). B- and T-cell responses to primary vaccination did not differ by age, sex, EDSS, or type of first vaccine. The duration between first vaccination and blood collection had a significant effect on the T-cell response to the S2 antigen pool (*p* < 0.012), i.e., the immune response to S2 was lower when more time had elapsed between vaccination and measurement.

### 3.3. Patient Characteristics in the Cohort after Booster Immunsation

A total of 305 patients were included in the evaluation after booster vaccination (Table 1). In this cohort, mean age was 47.2 years and 210 patients (68.9%) were female. The majority of patients had a diagnosis of relapsing-remitting MS (76.4%), whereas a progressive disease course was documented for 23.6%. Two-hundred-and-forty-eight patients (81.3%) received treatment with ocrelizumab, three (1.0%) with rituximab, two (0.7%) with ofatumumab, and fifty-two (17.0%) patients received no immunomodulatory treatment. Mean time period between booster vaccination and blood sampling was 52.4 (33.63) days (mean (SD)).

### 3.4. SARS-CoV-2-Specific T- and B-Cellular Immune Response after Booster Vaccination

**Figure 4:**
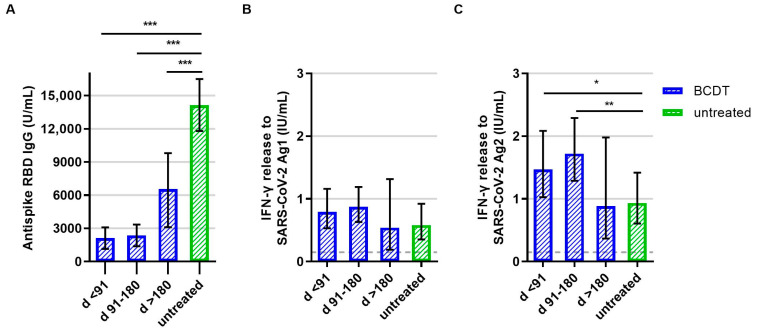
B- and T-cellular responses to booster vaccination in anti-CD20 depleted versus untreated patients (*n* = 305). B- and T-cell responses in patients with B-cell-depleting therapy (BCDT) (blue, *n* = 253) and untreated (green, *n* = 52) patients to the booster vaccination are presented. BCDT is further differentiated regarding timepoint of last BCDT treatment cycle and vaccination: <91 days (*n* = 87), between 91 and 180 days (*n* = 146) and >180 days before vaccination (*n* = 20). (**A**) presents the B-cell response, (**B**) the T-cell response to Ag1, and (**C**) the T-cell response to Ag2. Means with 95%CI are presented. The dashed line shows the cut-off for a positive T-cell response, defined at 0.015 IU/mL. For the B-cell response, the cut-off level is defined at 0.8 U/mL. Data were analyzed with generalized linear models with gamma distribution and log link function. Sex, age, Expanded Disability Status Scale, time between vaccination and blood sampling, confirmed previous COVID-19 infection, and treatment group served as fixed factors. For pairwise comparisons, contrast tests with Sidak correction were applied. Asterisks indicate level of statistical significance: * *p* < 0.05, ** *p* < 0.01, *** *p* < 0.001. (**A**): *p* < 0.001 for untreated compared to d >180, *p* = 0.000 for untreated compared to d 91–180, *p* = 0.000 for untreated compared to d < 91, (**C**): *p* = 0.034 for untreated compared to d < 91, *p* = 0.005 for untreated compared to d 91–180.

After booster vaccination, patients with an anti-CD20 therapy presented significantly lower anti-RBD antibody titer levels compared to patients without treatment (Figure 4A). As for T-cell responses, IFN-γ release to SARS-CoV-2 peptide pool 2 was significantly higher in anti-CD20-treated patients who had received the booster shot less than 180 days after their last treatment cycle compared to patients without treatment (Figure 4C). Concerning peptide pool 1, a similar trend was visible but the difference was not statistically significant (*p* = 0.090, Figure 4B).

Of the patients with anti-CD20 therapy, 42.3% had a positive SARS-CoV-2 antibody response and 85.0% of the patients with BCDT had a positive T-cell response after booster vaccination. All patients without therapy had a positive B-cell response after booster vaccination. Of the patients without therapy, 67.3%had a positive T-cell response (Figure 2).

Prior COVID-19 infection had a significant effect and increased SARS-CoV-2-specific antibody (*p* < 0.001) and T-cell response (*p* = 0.047 for peptide pool 1, *p* < 0.003 for peptide pool 2) after booster vaccination (Figure 3). B- and T-cell responses to booster vaccination did not differ by age and sex.

### 3.5. SARS-CoV-2-Specific T- and B-Cellular Immune Response at Follow Up

**Figure 5:**
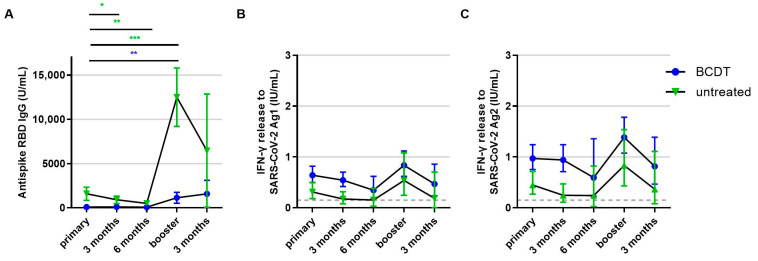
Development of B- and T-cellular responses during follow up in anti-CD20 depleted versus untreated patients (*n* = 218). B- and T-cell responses in patients with B-cell-depleting therapy (BCDT) (blue, *n* = 165) and untreated (green, *n* = 53) patients after vaccination and during follow up are presented (respective *n* for each time point: 218, 84, 10, 149, 36). (**A**) presents the B-cell response, (**B**) the T-cell response to Ag1, and (**C**) the T-cell response to Ag2. Means with 95%CI are presented. The dashed line shows the cut-off for a positive T-cell response, defined at 0.015 IU/mL. For the B-cell response, the cut-off is defined at 0.8 U/mL. Data were analyzed with generalized linear mixed models (GLMM) with gamma distribution and log link function. Sex, age, Expanded Disability Status Scale as measure of disability, time between vaccination and blood sampling, timepoint, type of vaccination, confirmed previous COVID-19 infection, treatment group, and the interaction of treatment group and timepoint served as fixed factors. For pairwise comparisons, contrast tests with Sidak correction were applied. Asterisks indicate level of statistical significance: * *p* < 0.05, ** *p* < 0.01, *** *p* < 0.001. (**A**): BCDT: *p* = 0.003 for primary compared to booster, untreated: *p* < 0.001 for primary compared to booster, *p* = 0.013 for primary compared to 3 months, *p* = 0.009 for primary compared to 6 months.

A subgroup analysis was performed to evaluate the development of the SARS-CoV-2-specific T- and B-cellular immune responses over time. Patients that presented a positive T- and/or B-cellular response to SARS-CoV-2 vaccination and no COVID-19 infection were scheduled for follow-up measurements (Figure 5).

The anti-RBD antibody titer of patients with anti-CD20 therapy remained below the titer of patients without therapy during the whole observation period. In contrast, the T-cell response of patients with anti-CD20 therapy was consistently higher than the T-cell response of untreated patients. Untreated pwMS exhibited a significant decrease in antibody levels both three and six months after primary vaccination, and antibody titers strongly increased upon booster vaccination. In anti-CD20-treated pwMS, there was no significant decrease in antibody levels after primary vaccination but antibodies increased upon booster vaccination. Antibody titers after the booster shot were significantly higher than titers after primary vaccination (Figure 5A). T-cell response presented a decreasing trend after primary and booster vaccination in both groups; however, this was not statistically significant (Figure 5B,C).

Trends in the longitudinal development of immune responses within each treatment group were similar when including all measured samples without pre-selection of the patients with an initially positive SARS-CoV-2-specific immune response. Statistical comparison of antibody titers after primary and booster vaccination for all patients including initially seronegative ones also showed significantly higher titers after booster compared to primary vaccination in untreated as well as anti-CD20-treated pwMS.

### 3.6. SARS-CoV-2-Specific T- and B-Cellular Immune Response in Patients with Therapy Switch to BCDT

The immune response of eleven patients receiving DMTs other than anti-CD20 therapy was measured after primary vaccination. At different timepoints after primary vaccination, these patients started BCDT and were on this therapy at the time of booster vaccination. In addition, there were five patients without therapy at primary vaccination that started anti-CD20 therapy before booster vaccination.

In this group, the B-cell response was higher compared to patients who received BCDT already at primary vaccination (Table 3). An increase after booster vaccination was also observed. All 16 patients were seropositive after primary and booster vaccination. After booster vaccination, the B-cell response in this group of patients was also above the titer of the patients that were vaccinated on BCDT (Table 3). Although the T-cell response was lower in the 16 patients vaccinated before the start of BCDT compared to patients on BCDT, all patients presented a positive T-cell response after primary and booster vaccination (Table 3).

## 4. Discussion

Immunotherapies carry the risk of an increased frequency and severity of infections. Protection induced by vaccination is hence of paramount importance for patients receiving these therapies. Therefore, we conducted a study to evaluate the long-term immune response to SARS-CoV-2 vaccination and infection in a cohort of pwMS with anti-CD20 treatment compared to no DMT. We found significant differences in the longitudinal development of antibody and T-cell responses to SARS-CoV-2 vaccination between anti-CD20-treated and untreated pwMS. As expected, antibody responses were significantly higher in the untreated group compared to the B-cell-depleted group after both primary and booster vaccination. However, T-cell responses were similar or, after the booster vaccination, even enhanced in anti-CD20-treated patients if the last treatment cycle had taken place less than 180 days before vaccination. These results are in line with previously published data [2,3,4,5,6,7,8,9,10,11,12,13,14,15,16,17]. As for the longitudinal development of immune responses, untreated pwMS showed a significant decrease in anti-SARS-CoV-2 antibody titers both three and six months after primary vaccination with a subsequent increase upon booster vaccination, which then remained stable for three months. In anti-CD20-treated pwMS, there was only a declining trend in antibody titers until six months after primary vaccination that did not reach statistical significance, but booster vaccination also led to significantly higher titers compared to primary vaccination. As reported in other studies, the lack of decline in antibody titers six months after primary vaccination in anti-CD20-treated pwMS was likely due to the initially low titers [3,23,24,25,26]. Concerning booster vaccination, our data correspond to several other studies showing a significant increase in antibody titers upon booster vaccination in anti-CD20-treated pwMS, though with a less steep increase compared to untreated pwMS [19,21,22,25,27,28]. In our cohort, 42.3% were seropositive after booster vaccination, corresponding to the range of the previously reported 33.3% and 85% [8,22,24,26,28,29,32].

T-cellular antiviral responses appeared to decrease in the months following primary vaccination and to increase upon booster vaccination in both untreated and anti-CD20-treated groups, but pairwise comparisons between the different timepoints did not reach statistical significance. These results correspond to another study that also did not find statistically significant differences in T-cell responses before and after booster vaccination [30]. Conversely, several other studies had reported a relevant decrease in virus-specific T-cell responses in the months after primary vaccination and a significant increase upon booster vaccination [16,18,28]. Overall, pwMS under anti-CD20 therapy developed normal or even enhanced T-cell responses to SARS-CoV-2 vaccination, which did not significantly decrease over time. These data suggest that at least a partially protective immune response against severe SARS-CoV-2 infections can be expected after vaccination.

Importantly, follow-up measurements were scheduled only in patients with a positive SARS-CoV-2-specific antibody or T-cell response in the initial sample. An immunizing event would be a vaccination or an infection that can lead to an activation of the immune system. Without these events, immune responses cannot be expected to change. We tried to limit the resulting bias by including only patients with initially positive immune responses in the statistical analysis of longitudinal responses. Trends were also similar when depicting all measured samples, indicating that the bias may indeed be limited. Our data show that booster vaccination also has a significant impact on the vaccine-specific immune response in anti-CD20-treated patients. This effect was robust in the pre-selected cohort of pwMS with an initially positive immune response, but also when including initially negative ones. Our data hence support the recommendation for a booster vaccination in pwMS under anti-CD20 treatment.

Our study shows a pronounced impact of COVID-19 infection on antibody and T-cell responses. Both responses were generally higher in patients who had a COVID-19 infection in addition to their vaccinations, which is in line with other published data in anti-CD20-treated patients as well as in the general population [11,25,33,34,35]. However, a few studies did not report a difference in antibody levels upon combined infection and vaccination [36,37]. In particular, hybrid immunity has been noted for its prominent effect on neutralizing SARS-CoV-2-specific antibodies, both in terms of their quantity and their neutralizing capacity with regard to a broad spectrum of variants [34]. These aspects were not studied in our analysis.

We used the commercial QuantiFERON interferon-γ release assay (IGRA) (Qiagen) for the analysis of T-cell responses. The advantage of this method is its easy applicability in routine diagnostics, enabling a faster analysis of a large number of samples due to the use of whole blood samples [11]. In a study involving 571 pwMS after SARS-CoV-2 vaccination, the comparability of the results obtained with the QuantiFERON assay with the results of ‘traditional’ T-cell assays was investigated. The QuantiFERON test correlated with activation-induced marker (AIM) flow cytometry after short-term whole blood culture as well as with interferon-γ enzyme-linked immune adsorbent spot (ELISpot) and AIM flow cytometric assays upon short-term peripheral blood mononuclear cell (PBMC) culture [38]. Further studies confirmed the concordance of IGRA results with flow cytometric analyses and ELISpot after SARS-CoV-2 vaccination and infection [39,40]. Of note, a different study reported that only 42% of kidney transplant recipients with a positive T-cell response in ELISpot analyses also had a positive QuantiFERON test [41]. Other data suggested that IL-2 is a more sensitive marker for SARS-CoV-2 spike-specific T-cells than IFN-γ [40]. Overall, due to the use of this test on whole blood instead of standardized cell counts, IGRAs may not be as sensitive as ‘traditional’ T-cell assays in immunocompromised individuals due to lymphopenia or the presence of medication in the sample [41]. However, our previous report analyzing T-cell responses to SARS-CoV-2 primary vaccination in anti-CD20-treated pwMS via QuantiFERON assay showed similar and partly even enhanced T-cell responses in the patients on immunotherapy compared to the ones without [11]. In summary, IGRAs seem to be a suitable method for the screening of T-cell responses in large cohorts but need to be complemented with more differentiated analyses for a more in-depth understanding of vaccination responses.

The most important limitation of our study is the previously discussed selection bias for the follow-up measurements. Our cohort was further not powered for the distinction of vaccination responses between different anti-CD20 treatment regimens. Our study also lacks data on the clinical efficacy of vaccination and on the severity of COVID-19 infections. COVID-19 infection status was assessed by positive RT-PCR results; however, asymptomatic and potentially not-tested patients may bias the study’s results. Moreover, the overlapping effects of previous DMTs cannot be fully ruled out even though we ensured a minimum interval of 28 days or more (depending on the respective DMT and its mechanism of action) between the last dose and the primary vaccination. A strength of our study is the comparably large number of patients included and the real-world setting, leading to a good generalizability of our results.

Overall, our data add to the existing knowledge about immune responses to vaccination against SARS-CoV-2 and COVID-19 infection. They support the recommendation for a booster vaccination in anti-CD20-treated pwMS and demonstrate an easy approach for the measurement of both anti-SARS-CoV-2 antibody and T-cell responses in clinical routine.

## Figures and Tables

**Table 1:** Characteristics of patients.

Analysis after	Primary Vaccination	Booster Vaccination
*n* (%)	368 (100)	305 (100)
Age years, mean (SD)	46.4 (11.9)	47.2 (12.2)
Female patients *n* (%)	257 (69.8)	210 (68.9)
Time between primary vaccination and blood sampling, days, mean (SD)	127.3 (64.09)	271.9 (48.03)
Time between booster vaccination and blood sampling, days, mean (SD)	-	52.4 (33.63)
Treatment duration until blood sampling, days, mean (SD)	915.8 (772.4)	831.7 (503.87)
Treatment		
Ocrelizumab treated, *n* (%)	285 (77.4)	248 (81.3)
Rituximab treated, *n* (%)	2 (0.5)	3 (1.0)
Ofatumumab treated, *n* (%)	2 (0.5)	2 (0.7)
Untreated *n* (%)	79 (21.5)	52 (17.0)
Disease course		
RRMS, *n* (%)	286 (77.7)	233 (76.4)
SPMS, *n* (%)	35 (9.5)	35 (11.5)
PPMS, *n* (%)	47 (12.8)	37 (12.1)
Vaccines		
BNT162b2 vaccine, *n* (%)	309 (84.0)	260 (85.2)
mRNA-1273 vaccine, *n* (%)	23 (6.3)	19 (6.2)
AZD1222 vaccine, *n* (%)	25 (6.8)	24 (7.9)
Ad26.COV2.S vaccine, *n* (%)	11 (3.0)	2 (0.7)

SD: standard deviation BCDT: B-cell-depleting therapy, RRMS: relapsing remitting multiple sclerosis, SPMS: secondary progressive multiple sclerosis, PPMS: primary progressive multiple sclerosis, BNT162b2 vaccine: Pfizer–BioNTech, mRNA vaccine, mRNA-1273 vaccine: Moderna—mRNA vaccine, AZD1222 vaccine: Oxford–AstraZeneca—viral vector vaccine, Ad26.COV2.S vaccine: Johnson&Johnson—viral vector vaccine.

**Table 2:** Distribution of patients with COVID-19 infection (*n* = 366).

	BCDT	Untreated
complete primary vaccination (*p* = 0.193)		
COVID-19 infection, *n* (%)	35 (12.1)	14 (18.2)
no COVID-19 infection, *n* (%)	254 (87.9)	63 (81.8)
booster vaccination (*p* = 0.999)		
COVID-19 infection, *n* (%)	43 (17.0)	9 (17.3)
no COVID-19 infection, *n* (%)	210 (83.0)	43 (82.7)

BCDT: B-cell-depleting therapy.

**Table 3:** SARS-CoV-2-specific B-cell and T-cell responses in patients before versus on BCDT at primary vaccination.

	After Primary Vaccination	After Booster Vaccination
Primary vaccination before BCDT		
Antispike RBD IgG (U/mL) (mean [95% CI])	550.8 [175.74–925.90]	3035.1 [−457.29–6527.49]
IFN-g release to SARS-CoV-2 Ag1 (IU/mL) (mean [95% CI])	0.28 [−0.01–0.56]	0.26 [0.04–0.49]
IFN-g release to SARS-CoV-2 Ag2 (IU/mL) (mean [95% CI])	0.33 [−0.12–0.78]	0.47 [−0.13–1.07];
Primary vaccination on BCDT		
Antispike RBD IgG (U/mL) (mean [95% CI])	95.3 [30.85–159.73]	1139.5 [524.04–1754.96]
IFN-g release to SARS-CoV-2 Ag1 (IU/mL) (mean [95% CI])	0.64 [0.50–0.82]	0.84 [0.62–1.12]
IFN-g release to SARS-CoV-2 Ag2 (IU/mL) (mean [95% CI])	0.97 [0.75–1.24]	1.39 [1.08–1.78]

BCDT: B-cell-depleting therapy.

## Data Availability

Data are available on request.
